# Patient Participation in Home Care: A Longitudinal Exploration of Experiencing, Refraining, and Losing Involvement

**DOI:** 10.1177/23779608251362652

**Published:** 2025-07-28

**Authors:** Ove Hellzén, Tove Mentsen Ness, Kari Ingstad, Mette Spliid Ludvigsen, Siri Andreassen Devik

**Affiliations:** 1 Department of Nursing Science, 621628 Mid-Sweden University, Sundsvall, Sweden; 2 Faculty of Nursing and Health Sciences, 1786 Nord University, Namsos, Norway; 3 Faculty of Nursing and Health Sciences, 1786 Nord University, Levanger, Norway; 4 Department of Clinical Medicine-Randers Regional Hospital, 1006 Aarhus University, Aarhus, Denmark; 5 Faculty of Nursing and Health Sciences, 1786 Nord University, Bodø, Norway; 6 Centre of Care Research, Mid-Norway, Faculty of Nursing and Health Sciences, 1786 Nord University, Namsos, Norway

**Keywords:** patient involvement, shared decision making, home nursing, healthcare professionals, older adults, family caregivers

## Abstract

**Background:**

Ensuring safe and personalized healthcare requires that patients have opportunities to express their concerns and influence their treatment decisions, which is a core value in healthcare. Such involvement is crucial for realizing the ideal of ageing in place. Despite efforts to increase patient participation, significant challenges persist, especially among older people with complex health needs.

**Aim:**

The aim of this study was to explore an older adult's experiences of patient participation within a care triad, as reflected in the interactions and perspectives of the older person, healthcare professionals, and a family caregiver in the Norwegian home-care context.

**Methods:**

This study employed a longitudinal single-case study with embedded units. Over 1 year, repeated interviews were performed with an older adult, his daughter, his general practitioner, and his responsible home-care nurse. Content analysis was used to analyze the data.

**Findings:**

The analysis resulted in three themes representing the participants’ experiences and points of view regarding the older adult's participation in care: *to experience participation*, *to refrain from participation*, and *to lose one's participation.*

**Conclusions:**

The findings indicate that the older adult desired involvement despite occasional reluctance and that his participation was affected by interactional and contextual factors. Professionals must realize that the starting point of patient involvement is the patient's perspective and understanding of care needs, which the professionals have a joint responsibility to meet.

## Introduction

To ensure patients perceive healthcare as safe and tailored to their situation and needs, care providers must ensure opportunities for patients to voice their concerns and influence decisions regarding their treatment and care (Carman et al., 2013). In Norway, as elsewhere in the world, the concept of patient participation is a core value in the healthcare system (Norwegian Ministry of Health and Care Services, 2019; Nolte et al., 2020). However, although considerable effort from both authorities and healthcare services to improve patient participation, major challenges remain, especially among older patients who are more vulnerable due to multimorbidity and the need for complex services across different sectors (Dyrstad et al., 2015; Pel-Little et al., 2021). A systematic review (Pel-Little et al., 2021) has documented barriers both in terms of predisposing factors (e.g., cognitive and physical impairments of older people), interactional factors (e.g., communication, roles, and support from informal caregivers) and organizational factors (e.g., organizational complexity and constraints).

## Review of Literature

Older adults with complex health needs are an increasing group of people who require health and care assistance in their own homes. Hospital stays are getting shorter and both advanced home care and frequent transitions between hospital and home challenge cooperation and involvement (Lilleheie et al., 2019). In Norway, home care comprises two services: (a) healthcare, which includes free-of-charge services related to personal hygiene, medication, and wound care and (b) practical assistance, such as food ordering, cleaning, and laundry help, which incurs a user fee based on income. According to Tuntland and Ness (2014), home rehabilitation has become a key strategy in Norwegian home care to enhance older adults’ resilience and quality of life, while reducing their reliance on public services. This approach uses empowerment strategies to motivate patients, aiming to improve functionality and foster independence in daily life, while the rehabilitation process emphasizes constant adaptation to patients’ priorities, with motivational work and active patient participation being fundamental (Tuntland & Ness, 2014).

The home is traditionally an arena where autonomy and participation are particularly emphasized in the interaction between the patient and healthcare professionals (Jacobs, 2019). However, older adults still encounter paternalistic attitudes and can feel like “guests in their own homes” when visited by healthcare professionals (Ferreira et al., 2015; Jarling et al., 2018). According to Johannessen and Steihaug (2019), care providers often define the patients’ needs themselves rather than spending time acquiring knowledge about patients’ real needs and wishes. This type of disregard also occurs during transitions between the hospital and home, where challenges with mutual communication and asymmetric relationships exist (Lilleheie et al., 2019). Angel and Frederiksen (2015) stated achieving an optimal level of patient participation is contingent upon establishing a framework that allows sufficient time for both patients and care professionals to foster relationships and exchange knowledge while also considering the inherent asymmetry in their relationship.

While contemporary healthcare embraces the idea of patient participation, a universally agreed-upon definition is lacking (Eldh et al., 2020). The concept of patient participation has been described based on the perspective of different professionals, such as nursing (Cahill, 1996; Nilsson et al., 2019) and medicine (Thompson, 2007). The medical understanding emphasizes decision-making processes and distinguishes between patient *involvement*, where the patient mainly receives information, and *participation*, which involves a certain degree of transfer of power and reciprocity between the patient and the doctor (Thompson, 2007). In a nursing context, the essence of patients’ participation is focused on learning, the caring relationship and reciprocity (Cahill, 1996; Nilsson et al., 2019). A term closely associated with patient participation is person-centred care, serving as both a pre-requisite and a benefit of participation (Søgaard et al., 2021). In the literature on person-centred care, patient empowerment and emancipation are the main aims (El-Alti et al., 2019). In Norwegian health legislation (Pasient- og brukerrettighetsloven [The Patient and User Rights Act], 1999/2020), patient participation is reflected as both a formal right and a therapeutic tool (Kettunen et al., 2003).

The complexity of the concept of patient participation makes it difficult to translate into a clinical context and the understanding varies across roles. Health professionals tend to define patient participation primarily in terms of involvement in decision making (Castro et al., 2016), whereas patients perceive it more broadly, encompassing the sharing of experiences and information, collaborative decision making and self-management (Eldh et al., 2010). Additionally, patients’ preferences for participation vary and are influenced by the type of healthcare interaction and their reason for seeking care (Årestedt et al., 2019).

The goal of participation also applies to family carers who play a pivotal role when older adults are cared for at home. Family caregivers desire to be involved in care teams and view their involvement as key to safeguarding the older adult's integrity and rights (Bragstad et al., 2014). However, the family's perspective remains largely unexplored (Ris et al., 2019).

The meaning of being involved in one's own treatment and care differs among individual older adults, healthcare professionals, and relatives (Sahlsten et al., 2008). The research in the field indicates that the various perspectives are most often examined separately and in the form of benefits or satisfaction with participation in cross-sectional studies (Riffin et al., 2021). Implementation of participation as a political ideal occurs primarily in the interaction between the person who receives help and the person who delivers it. This meeting can be a practical extension of society's democratic governance and is an arena that can provide deep and nuanced knowledge. The aim of this study was, therefore, to explore an older adult's experiences of patient participation within a care triad, as reflected in the interactions and perspectives of the older person, healthcare professionals, and a family caregiver in the Norwegian home care context.

## Method

### Study Design

A longitudinal single-case study with embedded units (Yin, 2018) was conducted to explore an older adult's experiences of patient participation within a care triad in home care. The design draws on three core features of case study methodology: uniqueness, embeddedness, and a longitudinal approach (Stake, 1995; Yin, 2018).

The case was selected for its intrinsic value in illustrating contrasts and complexity in the phenomenon of patient participation, making it particularly suited for an in-depth, contextual study rather than for generalization (Stake, 1995). It stood out among nine cases from a previous multiple-case study on interaction experiences of older care recipients, their relatives, and healthcare professionals in Norwegian municipal home care (Hellzén et al., 2024). Embedded units—involving different actors—enabled an exploration of how the phenomenon manifested from various perspectives, allowing for internal triangulation of findings (Yin, 2018). Rather than questioning the validity of the older adult's experience, this triangulation aimed to enrich its interpretation by situating it within a broader context informed by the perspectives of other actors involved in the same events. In the context of a care triad, participation is not only individually experienced, but co-constructed in interactions between the older adult, their relatives, and healthcare professionals. Capturing these interrelated perspectives is therefore essential to understand participation as a relational and negotiated phenomenon, rather than as a fixed or purely individual process. Triangulation thus served not only to enrich the data but to reflect the very nature of the phenomenon under investigation. This approach supports a rich understanding of the phenomenon within its natural context, where the boundaries between case and context are often blurred (Scholz, 2011; Stake, 1995; Yin, 2018). Finally, the longitudinal perspective was essential for capturing changes and continuities in participation over time, across different phases and from multiple viewpoints (Yin, 2018).

To ensure comprehensive reporting of all perspectives, we followed the COREQ checklist (Tong et al., 2007).

### Study Setting, Participants, and Data Collection

In the current study, the main unit of analysis was an 88-year-old man with multimorbidity and complex support needs, referred to by the pseudonym Ola, and his home context. Ola received home nursing care twice daily, mainly for help with medication administration and personal hygiene. Ola's case was selected from a previous multiple-case study (Hellzén et al., 2024). The sampling strategy in that study was based on convenience, with the following inclusion criteria: adults aged 65 years or older, hospitalized within the past 3 months, receiving home nursing care at least twice daily, and having the cognitive capacity to provide informed consent. Recruitment support was provided by home care service managers in municipalities in Central Norway. Older adults who consented also granted permission to share information about relatives and healthcare professionals who could be invited for interviews. Recruitment and data collection are described in more detail in Hellzén et al. (2024).

Ola's case was selected due to the clarity of his accounts of personal experiences and the contrasting perspectives provided by other actors, which together revealed tensions considered important for in-depth exploration. The case was also sufficiently information-rich to respond to the purpose of the study. It was considered unique within the larger study, standing out from the other eight cases due to the marked divergence in how participation was experienced and described by the involved actors.

The case was delineated on two levels: temporal and spatial (Creswell, 2013; Ridder, 2017). Temporally, the case covered a 1-year period, beginning when the patient was discharged from the hospital and started receiving home nursing care. Spatially, it referred not only to the patient's adaptation to the home environment following discharge, but also to how the participants positioned themselves in relation to each other and to the phenomenon of “participation.”

Other actors linked to Ola (main unit of analysis) included three subcases: his daughter, his general practitioner (GP), and his designated home-care nurse. We assigned Ola the main role, whereas the other participants were considered supporting actors, as their experiences were analyzed in relation to his. The daughter was 63 years old and lived approximately 20 miles away from her father but had almost daily telephone contact with him. The doctor was a 30-year-old woman, who worked as a GP in the municipality and had regular consultations with Ola in recent years. The home-care nurse was a 40-year-old woman, who was trained as a registered nurse and had 11 years of experience in home care in the municipality. She acted as Ola's primary contact and had a good overview of his medical history and the services he received.

The last author collected data from autumn 2020 to autumn 2021 through semistructured interviews: three with Ola, two with his daughter and responsible home-care nurse, and one with his GP (Table 1). Although the plan was to conduct two interviews with the GP, as was done with the daughter and the home-care nurse, it was not possible for the GP to set aside time for more than one interview. Due to infection control measures related to the COVID-19 pandemic, the interviews were conducted digitally (via Microsoft Teams telephony). The participants were asked to talk about: (i) the time after discharge from hospital, (ii) experiences with care from the healthcare services, and (iii) thoughts about involvement in care and treatment. The interviews lasted from 25 to 90 min, were audiorecorded and later transcribed verbatim.

**Table 1.** Summary of Data Collection Time Points.

Participants	Baseline	After 6 Months	After 12 Months
Patient (Ola)	x	x	x
Relative (Daughter)	x		x
Nurse in home care	x		x
GP	x		

GP= general practitioner.

### Analysis

Data were analyzed using an inductive data-driven approach within qualitative content analysis (Graneheim & Lundman, 2004; Graneheim et al., 2017), starting with several open readings of the texts to gain an expanded understanding. First, the text was read several times to understand the content and identify “meaning units” that corresponded to the purpose of the study. These units were condensed with the meaning core preserved, which provided the basis for the development of codes. The codes were abstracted, compared based on differences and similarities and sorted into subthemes and themes. According to Graneheim et al. (2017), the method supports a flexible analytical process that can range from descriptive, manifest content to deeper, interpretative analysis of latent meaning. The focus shifted back and forth between parts and the whole, with attention to how the essence of the meanings reflected different actors at different points in time. We ended up with three central experiential dimensions, or overarching themes, which did not lend themselves to natural subdivision. Therefore, we do not present subthemes in the description of the themes; instead, examples are provided in Table 2 to demonstrate the transparency and credibility of the analytic process. Thematic saturation was considered achieved based on the breadth of the collected data and the repetition of themes during the analysis stage (Rahimi & Khatooni, 2024; Saunders et al., 2018).

**Table 2.** Examples From the Analysis.

Meaning Unit	Code	Subtheme	Theme
*They help me with a shower once a week – it's supposed to be on Thursdays, but if I want, it can be changed to another day*… *it's very convenient like that. They are all very kind and accommodating.* (Ola, Int. 1)	Having a say	Availability of options	***To experience participation***
*I believe he is quite satisfied with the information he receives, and he also asks for it himself. He doesn’t just sit back and wait for someone to tell him something.* (Daughter, Int. 1)	Knows what he wants	He actively seeks help for his needs	
*He wondered if he could get a support brace for his back, but he didn’t know where to get one and asked if I could help him. So, we did it together. … I feel that we know each other well, and it's mostly been me who has arranged and organized the help he receives. And I don’t think I’ve arranged anything he hasn’t agreed to, no.* (Nurse, Int. 1)	Responds to his wishes	The nurse provides what he asks for	
*He needed fluid treatment, and then it was kind of a question of whether we should take him to the nursing home or give him the treatment at home… And he was involved in that decision. He wanted to have it at home.* (GP, Int. 1)	Asks for preferences	Accommodated the wish for treatment at home	
*The doctors have too many patients*… *and fifteen minutes isn’t enough. They don’t really want more than one question, you know. If you have pain in one foot, that's enough. There's no time to talk about anything else.* (Ola, Int. 2)	Resigned acceptance	Just as well to let the doctor decide	***To renounce participation***
*He is still struggling with poor balance*… *he mentioned that he would like to see a physiotherapist, you know, because he believes the problems stem from his neck. But in his hometown, there aren't many physiotherapists with available appointments, so he doesn’t get that help. That's a bit unfortunate.* (Daughter, Int. 2)	Physiotherapy isn’t an option	Accept things as they are	
*He has received a back brace for his back pain, his medications are being followed up, and overall, he has improved. But the dizziness… or imbalance, that could have many causes—it's something he has to live with. Still, he functions well… he is resourceful.* (Nurse, Int. 2)	It's as good as it gets	Coming to terms with reality	
*…I suffer from dizziness, and I have spoken to the nurses about it, but they say they can't help me. So, I'll try with the doctor*… *They accept that I have dizziness. I don’t.* (Ola Int. 3)	Fighting for my cause	They don't care what I say	***To lose participation***
*Yes, I actually think the services he receives are working well. He's fortunate as long as he's able to manage in his own home.* (Daughter, Int. 2)	The service is working well	He is lucky to still manage to live at home	
*His case is a prime example of how well things can be done* (the organisation and collaboration) *and how well it can turn out in the end. But that is also because he is so resourceful and has a strong support network.* (Nurse, Int. 2)	An excellent example of effective collaboration	Mission completed	
*He was diagnosed with diabetes, and he had some opinions about the medication*… *I do feel that he's well informed, I really do, and he does have a say in it, so to speak.* (GP, Int. 1)	Information is given	Informed enough to have a say	

GP= general practitioner.

The first author conducted the analysis, while the other authors acted as co-analysts. To increase reliability, we reflected on and discussed the codes, themes, and subthemes until we reached a consensus on the findings. The authors have clinical experience as registered nurses in home care, nursing homes, and hospitals, including specialist expertise in geriatrics, psychiatry, and sociology. Differences in skills and experience led to frequent discussions about the reasonableness of the interpretations.

## Findings

The analysis resulted in three themes: to experience participation, to refrain from participation and to lose one's participation. A synthesized result based on these themes and the participants’ aggregated opinions on Ola's involvement in his care is presented below.

### To Experience Participation

Until recently, Ola had been a relatively healthy and active older adult who lived with his wife in an assisted living apartment in a sparsely populated municipality. He took few medications, exercised in a swimming pool several times a week, and was socially active. After a sudden illness and hospitalization, his life changed significantly. He now needed a walker to get around and required daily home nursing care.

Ola felt involved in his care when given the opportunity to express his own needs and ask questions, which he emphasized was crucial to feeling involved in his care.

Yes, the healthcare professionals come morning and evening. Now, after I got home from the hospital, I told them they have to help me with the medicine until I can manage on my own. They listened …//… so it turned out the way I wanted… so now they take over and manage it.

As Ola explained, believing that the nurses listened to him made him feel involved in his care. His daughter was also satisfied that the nurses listened to her father and took over responsibility for the medication, which she believed was important for his safety. Ola felt that it should be self-evident that his views were valuable and, therefore, considered by the healthcare professionals. He described that being able to ask the healthcare professionals questions was essential for the quality of their interaction. However, he mentioned some issues required a nurse with a higher level of medical training, so it was important for him to be aware of who was visiting him and their formal role.

Yes, I have fallen… out in the corridor and hit my side. It went well. I came up on my own. I have told the nurse, so they know. I am very satisfied with the home service …//… probably know almost everyone now… There are both nurses and assistants who come. Yes, I see a difference between who are nurses and who are assistants. There are quite a few assistants, they are just as good and helpful as the nurses. I know who they are… the assistants don't have this care tag; they just have a name tag.

Ola also described that his motivation was related to the healthcare professionals’ level of commitment and this influenced how he interacted when meeting with the staff. He said it was important he met a healthcare professional who listened to him. His daughter said:

It is probably important that he is motivated… and wants to do as much as possible to get better… the feeling that he can influence his care is important to him. I think so, he is probably the one who insisted on getting these examinations that he is getting now. Obviously, if he had just sat down, not said anything… I think maybe not so much would have happened. Now he at least talks about his wishes.

In the interviews, Ola explicitly expressed the importance of being seen and respected as a person and being able to influence his care. He described and emphasized the importance of the healthcare professional setting aside sufficient time, having an open approach in the meeting and making a conscious effort to understand how he was feeling. In the interview, the responsible nurse talked about a meeting that she thought turned out well:

He told me that he was going to celebrate Christmas with his relatives, and he didn't want to measure his blood sugar that often… then I said I would check it. I spoke to the doctor… and we agreed that I could call him [Ola] back and tell him that he no longer needed to do the measurements anymore.

The GP recounted an episode when Ola needed intravenous fluid treatment, during which he was given the choice of receiving the treatment at home or at the nursing home. At his own request, Ola received the treatment at home. Ola often talks about how important it is for him to live at home, something both his daughter and the healthcare professionals are aware of.

### To Refrain From Participation

For various reasons, Ola sometimes accepted a lack of influence and involvement in his care. Most often these occasions were due to his state of health, and he emphasized that the handover of decision making was temporary. He expected that when the time was right—when his health permitted, and he was able—he would again be involved in his care.

The first time I was at the hospital, I was told that they had ordered home healthcare. They didn't ask what I thought before they did it. When I got in better shape, I took the medicine myself. Then they didn't come to my house very often. I must admit that my experiences with healthcare have made me a little sceptical of doctors and healthcare.

He also explained that sometimes he had to accept that he did not always know what the healthcare professionals were talking about. Although he did not always demand that he comprehend everything that, for example, the doctor said, he remarked that it was tiring that he always had to concentrate during these visits. Indeed, the doctor's visits took up a lot of his time, but he felt it was also important he be given time to explain to the doctor what he wanted. However, strict scheduling sometimes led to him being forced to accept the care that the doctor offered him instead of being able to actively influence the care.

Yes, sometimes when I'm at the doctor they ask repeatedly… and then they sit there and look at the computer and then… well, then the minutes go by and suddenly the time is up, and it's time to go. The minutes go by fast when you are at the doctor's office… you have to concentrate to remember to ask about everything you want answered… Most of the time I don't have time, so I might as well let them manage the appointment.

In Ola's opinion, refraining from participation meant showing compliance and doing as the healthcare professionals said. Ola emphasized that he usually assumed that the healthcare system was working as it should and that healthcare professionals had sufficient knowledge and commitment to provide quality care. Therefore, he believed that there could be a risk that problems might arise if he did not agree with the healthcare professionals. As the responsible nurse described:

He himself does not ask for help or information. The first time I met him he was crying, because he didn't want to disturb others and he didn't really want to go to the hospital… he wanted to be home with his wife and… so we had to push a little… //… It's not like he's demanding anything.

The nurse stated that she had coordinated much of Ola's care and was working to provide the assistive devices he had asked for. Ola struggled with balance but was training with a walker to improve. Nevertheless, she believed it might be a persistent issue he would have to adapt to.

### To Lose One's Participation

Although the nonparticipation described above was self-selected, situations where he was deprived of participation or not invited to participate at all were more painful. Ola described feeling overlooked and objectified as well as violated and insecure at these times because he was used to being listened to in his professional career. His dependence on care meant that he saw no alternative but to accept the situation. He explained:

Life has taught me that you don't complain about the person you depend on. Then, you must be careful.

Being deprived of participation was perceived as being treated as a “thing.” Being viewed as an object instead of a person led to difficulties in maintaining an image of oneself as an independent person in daily life.

I feel like I'm in very good shape, but what's bothering me now… is that after all these trips to the hospital, I've lost my balance. So, I am completely dependent on the walker. Both inside and outside. Although I should really have been to a physiotherapist. I suffer from dizziness, and I have spoken to the nurses about it, but they say they cannot help me. So, I will try with the doctor… They accept that I have dizziness. I don’t.

He also talked about how he experienced insecurity in his care based on the healthcare professionals’ actions. For example, he said that they did not always answer his questions, so he felt he could not always trust them with his care. From the GP's perspective, verbally communicating information to older adults can be challenging. She preferred to send written letters, noting that at a certain age, written information tends to be more effective. Nevertheless, she believed that Ola was generally well informed about his health condition and medications, and that he largely had the opportunity to express his own opinions.

Communication was also seen as key to ensuring participation, from the daughter's perspective. She emphasized that her father's contact with the home care services was most important. She was aware that her father repeatedly requested physiotherapy, but pointed out that there was little she could do about it. She believed he had to come to terms with the fact that access to such services was limited in his local area.

The responsible nurse also tried to foster communication through informal visits, which she believed he appreciated. Ola confirmed that he valued the contact with the nurse, but maintained his position regarding the ailments he experienced and felt were not being understood. He expressed a sense of helplessness:

Especially in the evenings, I often have headaches. My doctor says we must wait and see until we get an answer to the X-ray. And if they don't find anything there, they should try to get an X-ray of the neck. But I've already done it on both back and neck… it's in my journal. I don't understand, they should be able to see that.

The disagreement with healthcare professionals could evoke a sense that the power imbalance was being reinforced. Ola gave the impression of being disappointed that he no longer perceived himself as independent. He described:

No, they don't know what it is…//… Speaking of crystal sickness. I mean, maybe I should have gotten some physical therapy, but they don't hear… If they had listened to me, they would have sent me to a physiotherapist. I went to training before I got this and got better, but lately, I haven't been able to do it because I've been so dizzy.

However, the perception that he was not being listened to was not shared by his responsible nurse. In her opinion, their communication was optimal:

If you think about his commitment to healthcare, I think he's been there all the way. From the beginning he was afraid to disturb anyone and afraid to go to the hospital, I had to motivate him, persuade him with information. But now it has lightened up a bit… We have reduced the number of meetings, but we are still in close contact. I'd say it's worth it. I don't think anything could have been different. Because when he's worse, the supervision increases, and we adjust the time. It's not him calling us on the phone, it's us calling him… For him, it has worked perfectly.

## Discussion

The study investigates an older adult's experiences of patient participation within a care triad in home care and highlights shifts between active participation and periods of nonparticipation. Ola, his daughter and the professionals all described how he sometimes meets his needs to be involved, while other times he does not. Patient participation thus manifests as a continuum from low to high levels of involvement (Thompson, 2007), where none of the parties expect absolute involvement but may differ in their opinions of sufficient involvement and timing. Ola and his daughter explained that much of his level of participation was based on his initiative. The GP and the nurse described being satisfied with their involvement and execution of their respective fields. They seemed unaware that Ola was left with unanswered questions and confused as to why he was not being listened to regarding complaints of dizziness and neck pain. The impression was that, whereas Ola's preferences for participation were dynamic and fluctuated particularly in response to changes in his health condition, the other actors maintained a more static and passive stance, appearing generally content with the level of participation as it stood.

### Person-Centred Participation

Since patient participation is a subjective perception, the realization of the concept requires a person-centered approach (Søgaard et al., 2021). Healthcare professionals must remain open to the understanding that the patient may transition between being a “consumer” of care and a “storied being” seeking consultation from different specialists from time to time (Dahlberg et al., 2009). According to Dahlberg et al. (2009), healthcare professionals should provide care based on the patient's life world, instead of an emphasis on the patient's agency alone.

Ola's experiences reflected a desire to engage and feelings of contentment when he was able to ask questions, and the professionals actively listened. On the contrary, he felt overwhelmed and helpless in encounters (e.g., at the doctor's office) when he did not experience a facilitation of dialogue. At other times, he understood that his state of health or physical form required him to take a more passive position. Such experiences underline the need for healthcare professionals to understand the reasons for occasional nonparticipation, and to respect the complexity of older adults’ experiences in their care. While “constrained participation” may be either involuntary or voluntary and even desirable for the patient (Kristjánsson & Thórarinsdóttir, 2024), Ola's experiences show that preferences may shift over time. If participation is truly to be person-centered, healthcare professionals must use both time and empathy to discover and respond appropriately (Kristjansson & Thórarinsdóttir., 2024). An exploratory dialogue has proven to be just as important to patients as the opportunity to make choices (Thórarinsdóttir & Kristjánsson, 2014).

Patients might exhibit limited confidence in their abilities and resources, particularly in vulnerable situations and when their health condition is changing (Pel-Littel et al., 2021). Providers may deem a form of weak paternalism as the most appropriate approach to delivering care until the health situation is better understood and the patient becomes more capable of actively participating in their care (Fernández-Ballesteros et al., 2019). Further, facilitating patient participation may necessitate additional resources. In present scenario, the patient expressed a desire for extended time with a doctor and physiotherapy. Although the constraints of limited resources can pose challenges in accommodating such patient preferences (Pel-Littel et al., 2021), fostering effective communication, and embracing shared decision making between healthcare professionals and patients is key for enhancing patient involvement. This collaborative approach empowers patients to advocate for services based on their preferences.

### Power Imbalance

The inevitable asymmetry of some interactions between the patient and healthcare professionals affects the possibilities and experiences of participation (Martinsen, 2009). Kristensson Uggla (2020) identified the patient's threefold disadvantage: being at the bottom of the care hierarchy, being in existential vulnerability, and facing disadvantages due to limited knowledge about their health condition. In this study, the doctor and nurse often set the conditions for Ola's participation, which can be perceived as authoritarian. He was informed that his dizziness was attributed to crystal sickness, and although he doubted this diagnosis, it was challenging to go against professional expertise. Simply being informed is often not sufficient for perceived participation; it also requires that the patient is able to comprehend the information (Eldh et al., 2010). Overall, such experiences of not being listened to threatened his perception of participation.

In addition to exhausting encounters with medical authorities, Ola had to navigate power relationships with the home-care staff. Experiencing an imbalance in power with professionals affected Ola's involvement in every aspect of his care, which was illustrated by his reluctance to risk expressing his needs due to his dependency on the nurses for care. Accordingly, older adults often avoid expressing their opinions about their care to maintain a balanced relationship with healthcare professionals (Kristensen et al., 2017; Molina-Mula & Gallo-Estrada, 2020). Although Ola's daughter was involved in his care, she did not consider herself the main informant and felt her father had the ability and desire to convey his needs to the healthcare team. The daughter's statements in the interviews could be interpreted as reflecting a supportive yet observational role. She acknowledged that her father could express his wishes and needs but also maintained that he had to accept that some of these needs might be difficult for professionals to meet. The daughter in this case did not live in close geographical proximity to her father, which may have influenced her level of involvement. However, other research shows that family caregivers often wish to be more involved when older adults receive healthcare, despite the burden this may place on them (Kraun et al., 2022). As the literature supports, while relatives can be important to compensate for the difference in authority, healthcare professionals have an important responsibility to create trust and reciprocity to realize true involvement (Cahill, 1996; Nilsson et al., 2019; Thompson, 2007).

As discussed above, building trust and reciprocity is a key responsibility of healthcare professionals when aiming to involve older adults meaningfully, particularly in the presence of relational asymmetries. While relatives can help bridge certain gaps in authority and communication, these dynamics remain complex and context-dependent.

Against this backdrop, there is a clear need for further research on how participatory processes between older adults, their families, and professionals can be strengthened in practice.

Methodologies that actively engage care recipients—such as co-creative approaches—may be particularly well-suited to fostering more equitable power dynamics and enhancing user empowerment in home-care contexts (James & Buffel, 2023; Kraun et al., 2022).

### Multiple Perspectives

It is important to understand that both old adults and care professionals may face challenges in terms of patient involvement due to their attitudes, skills, and expectations. Successful patient participation is based on respectful interaction, the promotion of a positive caring relationship and effective communication between professionals and the individual. The fact that multiple professionals are often involved in the care of older individuals adds further complexity (Doornebosch et al., 2022). From the nurse's point of view, participation can mean planning for Ola to receive information about the health condition and the range of different services (Cahill, 1996; Nilsson et al., 2019), while the doctor can focus on matters that concern decision making about Ola's health and treatment (Thompson, 2007). Despite each provider's role in meeting the patients’ health needs, it is important to consider whether their approach to care is congruent with how the patient himself understands his situation and experiences being a participant in his treatment and care. The findings suggest that the other actors involved in Ola's case failed to acknowledge his disappointment regarding the lack of empowerment. They may not have considered that Ola's life had changed significantly, and that he was perhaps comparing his current situation to a previously fully independent life. The GP and the nurse seemed to assess the quality of his participation based on a different standard—namely, his frail condition following the hospital stay. The GP and the nurse—as well as his daughter—may have drawn on this backdrop, which could explain their satisfaction, perceiving him as empowered to the extent reasonably expected given his health and age.

### The Home Care Context

As much as patient participation is influenced by the people who are part of the collaboration (the patient, relatives, and various professionals), participation does not happen in a vacuum. Existing research suggests that active participation in care is limited when the focus is solely on tasks rather than building a relational connection (Millard et al., 2006; Terry & Coffey, 2019). Concerning home care, a significant transformation takes place when one's living space is transformed into a professional environment. This change disrupts the conventional rhythm of the home by introducing a structured, chronological schedule, effectively turning the home into a workplace. This transition involves a shift from a relaxed pace to a carefully organized schedule, where time optimization and delineation of activities are prioritized. This focus aligns with Rosa's (2017) concept of time acceleration, which means that time is seen as a valuable resource to be used efficiently. Consequently, increased emphasis is placed on different activities or tasks and less on social interaction. As discussed, for example, by Hellzén et al. (2024), the need to optimize time leads to a situation where the old adult and the health professional risk not having enough time to build relationships and share knowledge (Angel & Frederiksen, 2015). Thus, the fast pace of service delivery can undermine the very advantages typically associated with the home environment—a setting that is, by its nature, conducive to person-centred care and thereby to participation, but which may lose its significance under such time pressure.

### Strengths and Limitations

Siggelkow (2007) argued that a single-case study provides sufficient information to describe a phenomenon. In this study, conducting repeated interviews created a sense of trust between the researcher and participants, which facilitated data collection. The longitudinal approach also made it possible to capture different events related to the applied phenomenon and to interview multiple individuals with different experiences of the same situation, which gave a broader perspective.

Although generalizability is not the aim in qualitative research, the researchers sought to ensure transferability by providing rich, contextualized descriptions of the case, its setting, and the interactions between participants (Lincoln & Guba, 1985). As Hamel et al. (1993) suggest, a well-conducted case study allows readers to “see the global in the local”—that is, to identify broader patterns and dynamics through the lens of a specific, situated case. In this spirit, the analysis aimed to illuminate how individual experiences and microlevel interactions within one care triad may reflect larger relational and systemic dynamics in municipal home healthcare. The case, while unique, is presented in a way that invites reflection beyond its immediate context.

However, participants’ awareness of others being interviewed may have influenced the information shared. A varying number of interviews were conducted with the participants. According to the study design, the older adult was to be interviewed three times, and both the relatives and healthcare professionals twice. A limitation is that the GP was only able to allocate time for a single interview.

### Implications for Practice

The study highlights the need for home care services to facilitate patient participation through relationship-building and tailored communication. Interprofessional arenas for collaboration and the systematic involvement of family caregivers can strengthen the care triad and support the patient's engagement. It is particularly important to identify and follow up on signs of withdrawal, as these may indicate a need for increased support rather than reduced willingness to participate. Healthcare professionals must also be attentive to the fact that patients’ needs for participation may vary over the course of care. Meeting individuals’ varying needs for involvement requires organizational flexibility and deliberate allocation of resources.

## Conclusion

This study described how an older adult adapted to a predefined care system and sometimes relinquished his right to have his suffering alleviated, leading to a tension between commitment and passive nonparticipation. The findings show how different actors in the care triad understand and relate differently to the patient's participation. Moreover, the case revealed that a lack of participation was not necessarily due to a lack of interest from the older adult but rather to interactional and contextual limitations. Professionals must realize that patient involvement begins with the patient's perspective and understanding of care needs, which the professionals have a joint responsibility to meet, and the pre-requisite is open and mutual communication between all parties.

Moreover, the findings suggest that traditional care models may not always support meaningful participation, and that future research should explore participatory and co-creative approaches that better accommodate the complexity of user involvement in care. Methodologies that actively include older adults as collaborators—rather than solely as recipients—may offer more equitable frameworks for designing care processes that are truly responsive to their needs.

## Supplemental Material

sj-pdf-1-son-10.1177_23779608251362652 - Supplemental material for Patient Participation in Home Care: A Longitudinal Exploration of Experiencing, Refraining, and Losing Involvement Supplemental material, sj-pdf-1-son-10.1177_23779608251362652 for Patient Participation in Home Care: A Longitudinal Exploration of Experiencing, Refraining, and Losing Involvement by Ove Hellzén, Tove Mentsen Ness, Kari Ingstad, Mette Spliid Ludvigsen and Siri Andreassen Devik in SAGE Open Nursing
